# Levosimendan increases the phosphorylation state of phospholamban in the isolated human atrium

**DOI:** 10.1007/s00210-022-02348-7

**Published:** 2022-11-29

**Authors:** Lina Maria Rayo Abella, Robert Hoffmann, Joachim Neumann, Britt Hofmann, Ulrich Gergs

**Affiliations:** 1 Institute for Pharmacology and Toxicology, Medical Faculty, Martin Luther University Halle-Wittenberg, 06097 Halle, Germany; 2 Department of Cardiac Surgery, Mid-German Heart Center, University Hospital Halle, Halle (Saale), Germany

**Keywords:** Calcium sensitizer, Phosphodiesterase inhibitor, Inotropy, Chronotropy, Mouse atrial preparations, Human atrial preparations

## Abstract

Levosimendan (up to 10 µM) given alone failed to increase force of contraction in isolated electrically stimulated (1 Hz) left atrial (LA) preparations from wild-type mice. Only in the additional presence of 0.1 µM rolipram, an inhibitor of the activity of phosphodiesterase IV, levosimendan increased force of contraction in LA and increased the phosphorylation state of phospholamban at amino acid serine 16. Levosimendan alone increased the beating rate in isolated spontaneously beating right atrial preparations from mice and this effect was potentiated by rolipram. The positive inotropic and the positive chronotropic effects of levosimendan in mouse atrial preparations were attenuated by 10 µM propranolol. Finally, we studied the contractile effects of levosimendan in isolated electrically stimulated (1 Hz) right atrial preparations from the human atrium (HAP), obtained during cardiac surgery. We detected concentration-dependent positive inotropic effects of levosimendan alone that reached plateau at 1 µM levosimendan in HAP (*n* = 11). Levosimendan shortened time of tension relaxation in HAP. Cilostamide (1 µM), an inhibitor of phosphodiesterase III, or propranolol (10 µM) blocked the positive inotropic effect of levosimendan in HAP. Levosimendan (1 µM) alone increased in HAP the phosphorylation state of phospholamban. In conclusion, we present evidence that levosimendan acts via phosphodiesterase III inhibition in the human atrium leading to phospholamban phosphorylation and thus explaining the positive inotropic effects of levosimendan in HAP.

**Supplementary Information:**

The online version contains supplementary material available at 10.1007/s00210-022-02348-7.

## Introduction

**Fig. 1:**
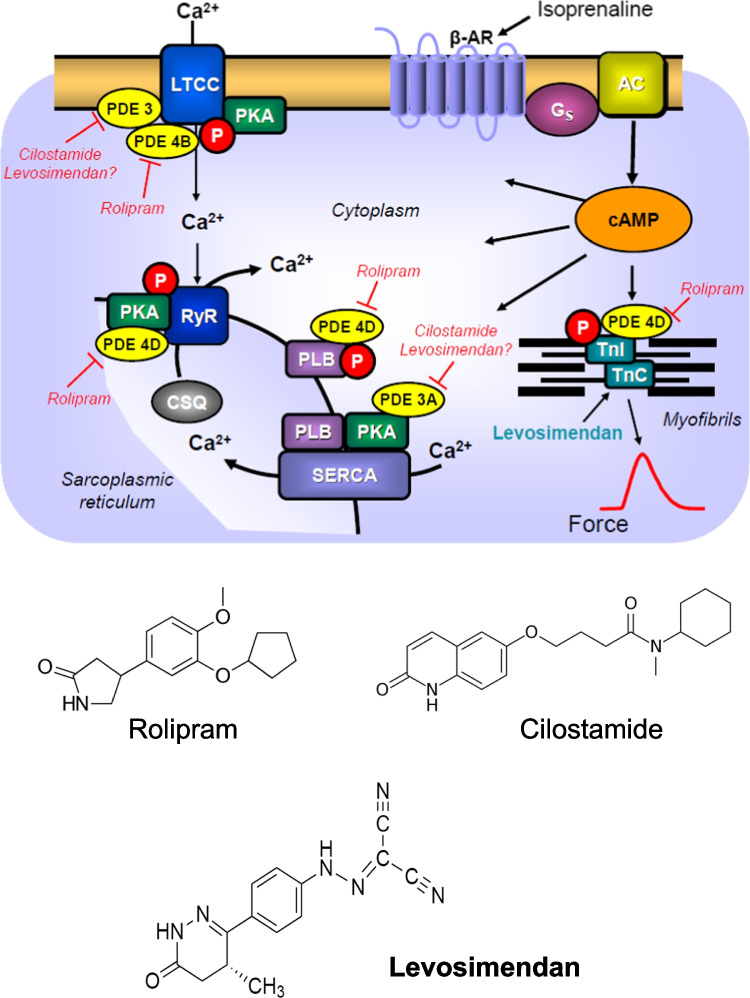
Scheme: Potential mechanism(s) of action of levosimendan in the human and mouse cardiomyocytes. Stimulation of the activity of β-adrenoceptors (β-AR) by endogenous noradrenaline or exogenous isoprenaline leads via stimulatory GTP-binding proteins (G_s_) to an increase of adenylyl cyclase (AC) activity. Adenylyl cyclase increases the formation of 3′,5′-cyclic adenosine monophosphate (cAMP) that stimulates cAMP-protein kinase (PKA). PKA phosphorylates and thus activates phospholamban (PLB) at the amino acid serine 16, the inhibitory subunit of troponin (TnI), the ryanodine receptor (RYR) and the L-type calcium channel (LTCC). The formed cAMP can be degraded to inactive 5′-AMP and pyrophosphate by isoenzymes of the phosphodiesterase family of proteins (PDE). Cilostamide and rolipram (bottom: structural formulae) inactivate phosphodiesterases 3 and 4, respectively. The β-adrenoceptor is blocked by β-adrenoceptor blockers like timolol or propranolol. Calcium cations (Ca^2+^) are stored on calsequestrin (CSQ) in the sarcoplasmic reticulum and are released via RYR from the sarcoplasmic reticulum (SR). These released calcium cations bind to troponin C on thin myofilaments, and as a result, systolic force is augmented. Typical calcium sensitizers like EMD57033 act by generating at a given level of free Ca^2+^ more force of contraction. In cardiac diastole, Ca^2+^ concentrations fall because Ca^2+^ is pumped into the SR via the SR calcium ATPase (SERCA). The activity of SERCA is increased when phospholamban is phosphorylated on amino acid serine 16. Levosimendan (bottom: structural formula) might act via increasing the sensitivity for calcium cations of troponin C in the thin myofilaments or might inhibit phosphodiesterases in the heart

Chronic and acute heart failure can lead to death. One option to treat heart failure remains to use positive inotropic drugs. In animal experiments, phosphodiesterase III inhibitors (scheme in Fig. 1) potently and effectively augmented force of contraction and beating rate (reviewed in Schmitz et al. 1989, 1992; Scholz and Meyer 1986). However, using failing human ventricular tissue, it turned out that phosphodiesterase III inhibitors had lost their efficacy to elevate force as heart failure worsened (Feldman et al. 1987; Leyen et al. 1991) (Fig. 1). This loss of the inotropic effects of phosphodiesterase III inhibitors in human hearts was explained by a reduced production of 3′,5′-cyclic adenosine monophosphate (cAMP) in the failing human heart (Danielsen et al. 1989; Feldman et al. 1987) (Fig. 1). Indeed, our group reported that cAMP levels in left ventricular muscle strips of failing human hearts were lower under basal conditions and increase less after β-adrenergic stimulation than those in nonfailing control samples (Danielsen et al. 1989). We presented also evidence that an increase in the expression of inhibitory alpha subunits of GTP-binding proteins might contribute to this diminished contractile response to β-adrenergic stimulation in failing human ventricular preparations by attenuating the stimulation of adenylyl cyclase activity (Neumann et al. 1988). Moreover, an increased expression of phosphodiesterases was reported in failing human hearts and might likewise contribute to the attenuation of inotropic effects of phosphodiesterase inhibitors in these failing hearts (Abi-Gerges et al. 2009; Sucharov et al. 2019).

In a seminal trial, more heart failure patients died (mostly from cardiac arrhythmias), who were treated with milrinone, a drug that inhibits the phosphodiesterase III than under placebo treatment (Packer et al. 1991). Hence, new mechanisms of inotropic action independent of an elevation of Ca^2+^ levels in the cytosol of cardiomyocytes (Fig. 1) were sought for. One such approach was the development of so-called calcium sensitizers (Rüegg et al. 1984; Ventura et al. 1992), like levosimendan (Haikala 1995, 1997) (structural formula in Fig. 1 bottom), CGP 48,506 (Neumann et al. 1996; Zimmermann et al. 1996), or EMD57033 (Neumann et al. 1995; Uhlmann et al. 1995). Calcium sensitizers raised the affinity of myofilaments for calcium cations. Levosimendan elevated force of contraction in guinea pig papillary muscles with an EC_50_ value (half maximally effective drug concentration) of 50 nM but levosimendan also potently reduced the activity of phosphodiesterase III (Kaheinen et al. 2006). However, based on other in vitro data (binding to a troponin, action in skinned cardiac fibers), levosimendan was not regarded by the developing pharmaceutical company as a phosphodiesterase inhibitor but as a calcium sensitizer (Haikala 1995). In contrast to this view, we and others reported in guinea pig cardiac preparations findings that were hard to reconcile with levosimendan being solely or relevantly a calcium sensitizer (Endoh 2015): levosimendan accelerated cardiac relaxation in the guinea pig heart (Bokník et al. 1997), levosimendan exerted a positive chronotropic effect in spontaneously beating right atrial preparations from guinea pig hearts (Bokník et al. 1997), levosimendan elevated cAMP concentrations, increased the phosphorylation state of phospholamban, the inhibitory subunit of troponin and C-protein and enhanced the current through the L-type calcium channel in cardiomyocytes from guinea pig ventricles (Bokník et al. 1997; Virág et al. 1996). In contrast, a pure calcium sensitizer, namely CGP 48,506, prolonged cardiac relaxation time and did not increase cAMP content nor phospholamban phosphorylation in guinea pig cardiac preparations (Zimmermann et al. 1996, 1998).

Others found in failing human ventricular muscle strips that while levosimendan could increase force of contraction, levosimendan could not significantly increase Ca^2+^ transients, which is a behavior expected in pure calcium sensitizers (Hasenfuss et al. 1998). However, reading carefully this paper, in muscle strips where force of contraction was maximally increased, also calcium transients were augmented, concurring with the assumption that levosimendan acted as a phosphodiesterase inhibitor also in humans under certain conditions (Hasenfuss et al. 1998). Several years later, the phosphodiesterase III inhibitor cilostamide nullified any positive inotropic effect of levosimendan in ventricular muscle strips from failing human hearts (Orstavik et al. 2014). This observation suggested to the authors that the positive inotropic effect of levosimendan was simply due to inhibition of phosphodiesterase III in the failing human ventricle (Orstavik et al. 2014). Their conclusion was supported by their finding that timolol reversed the positive inotropic effect of levosimendan in human ventricular preparations (Orstavik et al. 2014). This was explained by these authors in the following way: normally endogenous noradrenaline stimulates β-adrenoceptors (Fig. 1). This action of noradrenaline can be antagonized by, e.g., timolol or propranolol, and if cAMP were not produced, a phosphodiesterase inhibitor could not booster the concentration of cAMP in cardiomyocytes, and therefore, no positive inotropic effect of levosimendan could develop (Orstavik et al. 2014).

However, the effect of levosimendan in human heart on phospholamban phosphorylation has not yet been studied. Hence, we started this project. Since we have had no access to ventricular samples, we studied the effect of levosimendan on contractile function in isolated electrically paced human right atrial preparations in the absence and presence of known phosphodiesterase inhibitors. We also studied the effect of levosimendan on phospholamban phosphorylation in human atrial preparations. For comparison, we performed similar experiments on cardiac atrial preparations from mice. These mouse data have the potential benefit to study the effect of levosimendan on sinus node function in isolated preparations which is not readily feasible in human hearts. We deem our data clinically important to stratify which patients might benefit or may be harmed by levosimendan treatment and might therefore enter clinical guidelines (Maack et al. 2019; Papp et al. 2020).

## Materials and methods

### Contractile studies in mice

In brief, wild-type mice were sacrificed, the thorax was opened, and the heart was mobilized and cut from the ascending aorta to make sure the right atrium was not damaged. Then, the whole heart was transferred to a dissection chamber filled with gassed Tyrode’s solution at room temperature. Right or left atrial preparations were isolated and mounted in organ baths as described previously by our group (Gergs et al. 2013, 2017, 2019b; Neumann et al. 2003). Force was detected under isometric conditions, amplified and fed into a digitizer and quantified with commercial software (LabChart 8, ADInstruments, Spechbach, Germany).

### Contraction studies in human atrium

These experiments were performed as reported repeatedly (Gergs et al. 2009; Neumann et al. 2021a, b). In brief, during cardiac surgery, at the site where the cannula for extracorporeal circulation entered the heart, small muscle strips were obtained from the right atrium. Patients were aged between 47 and 74 years. Medication included acetylsalicylic acid, nitrates, diuretics, β-adrenoceptor blockers, and anticoagulants. Atrial trabeculae were dissected and mounted in an organ bath and electrically stimulated (1 Hz) and processed like mouse preparations (see above).

### Western blotting

The process of sample homogenization, protein concentration measurement, electrophoresis, antibodies incubation, and signal quantification were performed following our previously published protocols with slight modifications (Boknik et al. 2018; Gergs et al. 2009, 2019a). Electrophoresis was performed in Novex™ 4–12% “Tris–Glycine Plus Midi Protein Gels” (Invitrogen, Thermo Fisher Scientific, Waltham, MA, USA). The run was performed at 4 °C for approximately 1 h at 120 V in the “NuPAGE MES SDS Running Buffer” (Thermo Fisher Scientific, Waltham, MA, USA) using a XCell4 SureLock™ Midi-Cell chamber (Life Technologies by Thermo Fisher Scientific, Waltham, MA, USA). Protein transfer into membranes (Amersham™ Protran, GE Healthcare, Chicago, IL, USA) was performed at 2 A for 2 h at 4 °C. Membrane blocking for 1 h at room temperature was followed by overnight incubation at 4 °C with the primary antibody for serine 16—phosphorylated phospholamban (1:5000; catalogue number: A010-12AP; PLB Ser16; Badrilla, Leeds, UK), SERCA2 ATPase (1:20,000 for human samples and 1:10,000 for mice samples; catalogue number: ab2861; abcam, Cambridge, UK), phospho-troponin I (1:5000 for human samples and 1:10,000 for mice samples; catalogue number: 4004; Ser23/24; cell signaling technology, Leiden, The Netherlands), while calsequestrin antibody was used as loading control (1:20,000, product number: ab3516; abcam, Cambridge, UK). Visualization of the signals was performed by using chemiluminescent HRP substrate (Immobilon™ Western, Millipore, Merck; Darmstadt, Germany) and a digital imaging system (ImageQuant™ LAS 4000; GE Healthcare, Chicago, IL, USA).

### Data analysis

Data were treated as in most our previous studies (Gergs et al. 2019b; Neumann et al. 2021a, b). Shown are means ± standard error of the mean. Statistical significance was estimated by Student’s *t*-test or analysis of variance (ANOVA) if appropriate. A *p*-value of less than 0.05 was considered significant. Experimental data for agonist-induced positive inotropic and chronotropic effects were analyzed by fitting sigmoidal curves to the experimental data with GraphPad Prism 5.0 (GraphPad Software, San Diego, CA, USA). All other statistical analyses were performed as indicated in the figures and tables.

### Drugs and materials

(-)-Isoprenaline ( +)-bitartrate, rolipram, propranolol, and cilostamide were purchased from Sigma-Aldrich (Taufkirchen, Germany). Levosimendan was from Biozol (Munich, Germany). All other chemicals were of the highest purity grade commercially available. Deionized water was used throughout the experiments. Stock solutions were freshly prepared daily.

## Results

### Mouse studies

#### Force of contraction in left atrial preparations

**Fig. 2:**
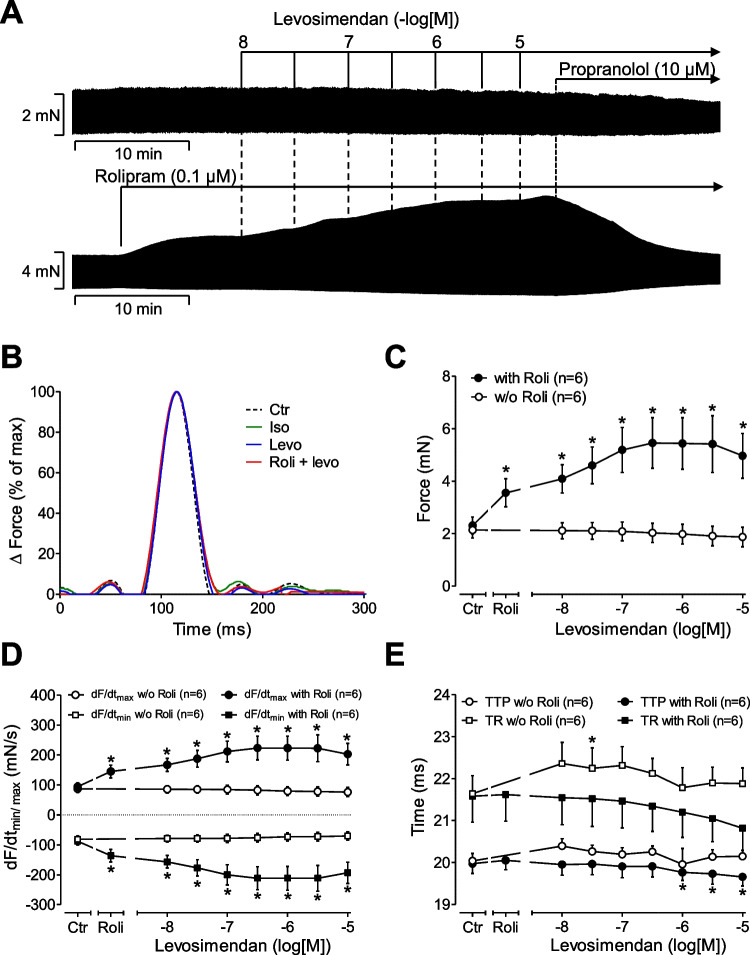
Positive inotropic effects of levosimendan in mouse atrium only in the presence of rolipram. (**A**) Original recordings**:** Levosimendan ((-)-(R)-[[4-(1,4,5,6-tetrahydro-4-methyl-6-oxo-3-pyridazinyl)phenyl]hydrazono]propane-dinitrile) (10 nM to 10 µM) was cumulative applied alone (**A**, top) or the presence 0.1 µM rolipram ((RS)-4-(3-cyclopentyloxy-4-methoxy-phenyl)pyrrolidin-2-on) (**A**, bottom). Rolipram was used to inhibit the activity of phosphodiesterase IV. The positive inotropic effect of levosimendan in presence of rolipram was abrogated by subsequently applied 10 µM propranolol. Vertical bars indicate force in millinewton (mN). Horizontal bars indicate time in minutes (min). (**B**) Effects of isoprenaline (Iso: 1 µM), levosimendan (Levo: 10 µM), or both (Roli + Levo) in single high-speed contractions compared to control conditions (no drug addition: Ctr). Please note that we normalized in the ordinate the maximum developed force of contraction under all conditions as 100% in order to facilitate detection of changes in contraction and relaxation times (abscissa in milliseconds (ms)). Concentration-dependent effects of increasing concentrations of levosimendan on force of contraction, or rate of tension or times of contraction in electrically stimulated left atrial preparations of mice are summarized in **C**, **D**, or **E**. Levosimendan was cumulatively applied alone (open circles) or after addition of rolipram (closed circles) to the organ bath. See (**A**) for details of drug addition and incubation times. Ordinates indicate the effect of levosimendan on force of contraction in millinewton (mN, **C**), on rate of tension development (in mN/s: **D**), on rate tension of relaxation (mN/s: **D**), on time to peak tension in milliseconds (ms: **E**), and on time of relaxation (ms: **E**) in electrically stimulated (1 Hz) isolated left atrial preparations from mice. Abscissae: negative decadic logarithm of the concentrations of levosimendan. *p < 0.05 versus Ctr (*t*-test). Numbers in brackets indicate the number of experiments

Levosimendan cumulatively applied, when given alone, from 10 nM to 10 µM (the highest concentration tested in this study) failed to increase force of contraction in paced (1 Hz) left atrial preparations of mouse hearts compared to control conditions (levosimendan alone: Fig. 2A). However, when 0.1 µM rolipram (a phosphodiesterase IV inhibitor) was given first, rolipram itself increased force of contraction, and additionally applied levosimendan increased force of contraction further, in left atrial preparations from mice. It might be worth mentioning that we first used 1 µM rolipram as 1 µM rolipram was used by others in rat ventricular preparations before they added levosimendan (Orstavik et al. 2014). However, 1 µM rolipram raised force of contraction in LA much higher than 0.1 µM rolipram and to such an extent that additionally applied levosimendan failed to increase force of contraction in LA (data not shown). For that reason, we switched here to 0.1 µM rolipram in LA. The positive inotropic effects of rolipram alone or levosimendan and rolipram were abrogated by subsequently applied 10 µM propranolol (Fig. 2A). The effects of levosimendan in the presence of rolipram were time-dependent and concentration (10 nM to 10 µM)-dependent (Fig. 2A). The positive inotropic effect reached its maximum at 1 µM levosimendan (Fig. 2A). However, subsequently applied 10 µM isoprenaline increased force of contraction in LA, further indicating that levosimendan under our experimental conditions is less effective than isoprenaline to raise force of contraction (data not shown). Interestingly, 1 µM cilostamide, a PDE III inhibitor, that effectively raised force of contraction in human atrial samples (vide infra) failed to raise force of contraction in LA (data not shown, reported before: Galindo-Tovar and Kaumann 2008; Neumann et al. 2019), consistent with a lower expression of PDE III relative to PDE IV in the mouse heart compared to the human heart (for review, see, e.g., table 2 in Neumann et al. 2019). Moreover, in the presence of 1 µM cilostamide, levosimendan failed to raise force of contraction in LA (data not shown), suggesting that PDE IV and not PDE III inhibition is necessary to unveil a positive inotropic effect of levosimendan in LA. When the maximally effective concentration of levosimendan (1 µM) was applied in the presence of 0.1 µM rolipram, then additionally applied 10 µM of EMD 57,033 ((R)-5-(1-(3,4-dimethoxybenzoyl)-1,2,3,4-tetrahydroquinolin-6-yl)-6-methyl-3,6-dihydro-2H-1,3,4-thiadiazin-2-one), a calcium sensitizer, increased force of contraction further (data not shown), suggesting that levosimendan does not act here as pure calcium sensitizer, because if that were the case, EMD 57,033 would be expected not to increase force any further than levosimendan in the presence of rolipram.

These data underscore that the presence of rolipram is necessary for levosimendan to elicit a positive inotropic effect in the mouse left atrium, like in the rat papillary muscle (Orstavik et al. 2014). Levosimendan increased the rate of tension development (Fig. 2D) and elevated absolute values of the rate of tension relaxation (Fig. 2D). Levosimendan also shortened time of tension relaxation and time to peak tension (Fig. 2E).

#### Force of contraction in right atrial preparations

**Fig. 3:**
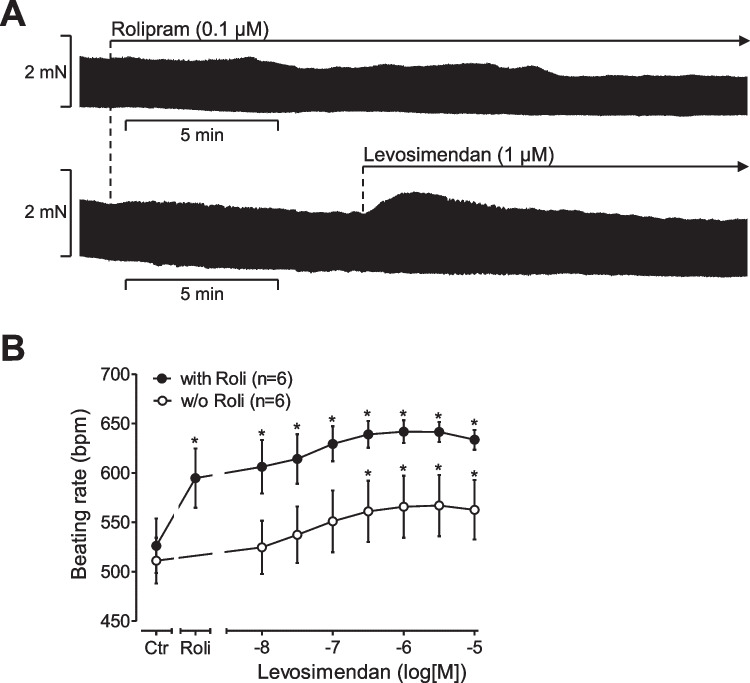
Positive chronotropic effect of levosimendan in the mouse atrium in the presence of rolipram. Original recording and summarized data. Levosimendan has a positive chronotropic effect in the mouse heart that is potentiated by the phosphodiesterase IV inhibitor rolipram. Ordinates in (**A**) indicate the effect of levosimendan on force of contraction or in beats per minute (**B**: bpm) in isolated spontaneously beating right atrial preparations from mouse hearts. Horizontal bars in (**A**) indicate time in minutes (min). Vertical bars in (**A**) indicate force of contraction in millinewton (mN). *p < 0.05 versus Ctr (*t*-test). Numbers in brackets indicate the number of experiments

One can discriminate between the initial phase after drug application and the steady state after drug addition (Fig. 3A). Using this definition, levosimendan alone does not affect either phase for force of contraction in spontaneously beating right atrial preparations (Fig. 3A). However, in the presence of rolipram (0.1 µM rolipram), levosimendan exerted a transient positive inotropic effect in the first phase of muscle contraction in right atrial preparations (Fig. 3A).

#### Beating rate in right atrial preparations

In the additional presence of 0.1 µM rolipram (Roli), which exerted a positive chronotropic effect of its own (Fig. 3B), levosimendan led to a concentration- and time-dependent positive chronotropic effect (Fig. 3B). The positive chronotropic effect of 10 µM levosimendan in the presence of 0.1 µM rolipram was blocked by additionally applied 10 µM propranolol (data not shown).

#### Phosphorylation in mouse atrial preparations

**Fig. 4:**
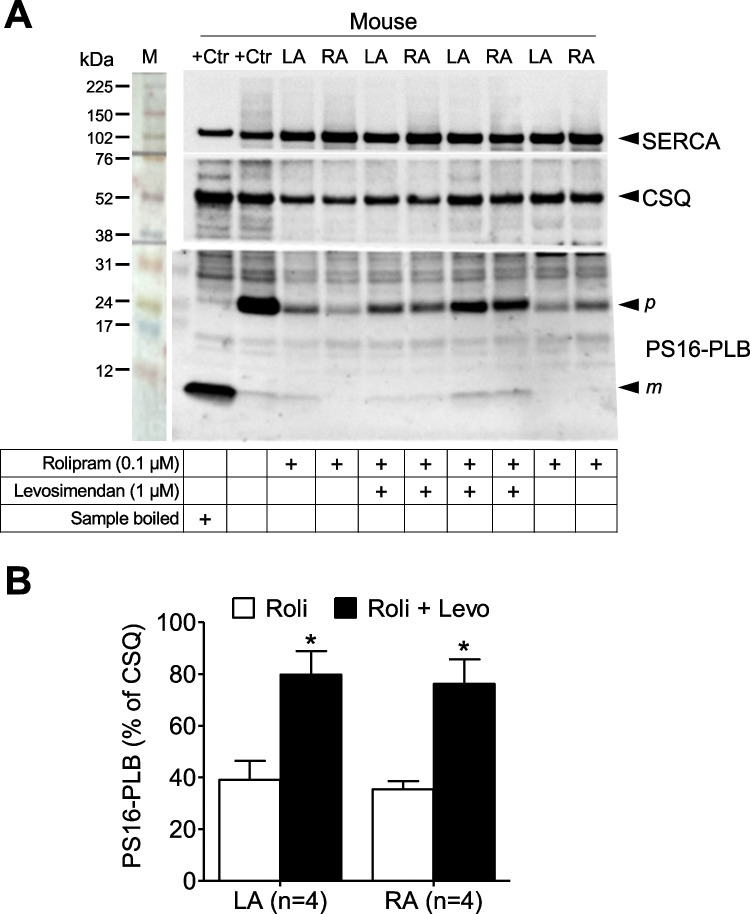
Levosimendan increases phospholamban phosphorylation in the mouse atrium only in the presence of rolipram. (**A**) The panel depicts a typical Western blot of electrically stimulated left atrial preparations (LA) or spontaneously beating right atrial preparations (RA) from mouse heart. Horizontal arrows indicate the apparent molecular weight in kilodalton (kDa) of phospholamban phosphorylated at serine 16 (PS16-PLB) or calsequestrin (CSQ), used as a loading control or sarcoplasmic calcium ATPase (SERCA: compare scheme in Fig. 1 for subcellular localization). Samples were treated with 1 µM levosimendan alone, 0.1 µM rolipram alone, or 1 µM levosimendan and 0.1 µM rolipram (Ro) combined (as indicated by + in the respective lanes). Apparent molecular weight standards are depicted in the left-hand first lane. “Boiled” means that a mouse LA sample was treated with 1 µM isoprenaline as a positive control (+ Ctr) for phosphorylation of phospholamban, and then loaded to the gel either after boiling (second lane from the left) or unboiled (third lane from the left). The typical mobility change for phospholamban is seen when comparing the high (p, pentameric) or low (m, monomeric) molecular weight form of phospholamban. (**B**) Quantification of Western blots for PS16-PLB normalized to the CSQ signal as loading control. *p < 0.05 versus rolipram (1-way ANOVA). Numbers in brackets indicate the number of experiments

Levosimendan at 1 µM increased the phosphorylation state of phospholamban at serine 16 in the presence but not in the absence of rolipram in contracting left atrial (LA) and right atrial (RA) preparations from the mouse heart (Fig. 4). Rolipram (0.1 µM) alone slightly increased the phosphorylation state of phospholamban in beating left or right atrial preparations (Fig. 4). However, in the additional presence of levosimendan phosphorylation of phospholamban greatly increased in both LA and RA (Fig. 4).

### Human studies

#### Force of contraction in isolated human atrial preparations

**Fig. 5:**
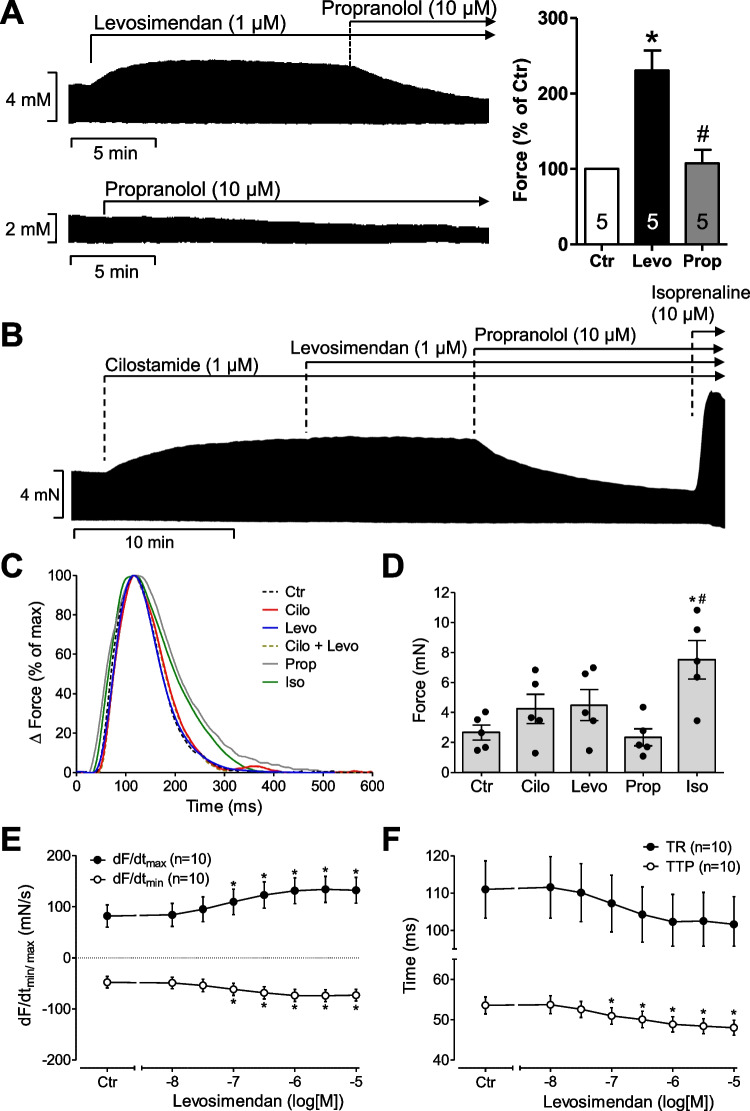
Inotropic effects of levosimendan. (**A**) Original recording of the inotropic effect of levosimendan (Levo, 1 µM) and its inhibition by propranolol (Prop, 10 µM) (upper recording). The lower recording shows a propranolol control, and on the right side, the quantification of force of contraction of samples from five patients is shown. Data are normalized to control conditions (no drug addition, Ctr). *p < 0.05 versus Ctr; ^#^*p* < 0.05 versus Levo (1-way ANOVA, *n* = 5). (**B**) The positive inotropic effect of levosimendan does not necessitate the concomitant inhibition of phosphodiesterases and is abrogated in the concomitant inhibition of phosphodiesterase III by 1 µM cilostamide in electrically stimulated muscle strips from human right atrium. The positive inotropic effects were abrogated by 10 µM propranolol. Subsequent application of a very high concentration of isoprenaline (10 µM) surmounted the effect of propranolol, indicating that the sample was properly contracting and that the efficacy of 10 µM isoprenaline was superior to that of 1 µM levosimendan. Vertical bars indicate force in millinewton (mN). Horizontal bars indicate time in minutes (min). (**C**) Typical sweeps of muscle contraction at high temporal resolution are plotted. Samples were incubated with 1 µM cilostamide alone (Cilo) or 1 µM levosimendan (Levo) or in the presence of both (Cilo + Levo) or in the presence of additional 10 µM propranolol (Prop) or isoprenaline (Iso) as in (**B**). (**D**) Mean values of force of contraction and SEM (*n* = 5) are presented by combining several experiments, as seen in the lower recording of (**A**). *p < 0.05 versus control conditions (no drug addition, Ctr); ^#^*p* < 0.05 versus propranolol (Prop) (1-way ANOVA, *n* = 5). (**E**, **F**) The concentration-dependent effects of cumulatively applied levosimendan are plotted for the first derivative of force in (**E**) and for time to peak tension (TTP) or time of relaxation (TR) in (**F**). Numbers in brackets indicate numbers of human muscle strips studied. *p < 0.05 versus control conditions (no drug addition, Ctr, *t*-test)

Cumulatively applied levosimendan alone exerted a concentration-dependent positive inotropic effect (Fig. 5D). In contrast, while 1 µM cilostamide increased force of contraction in human atrial preparations (Fig. 5A), cilostamide impaired any positive inotropic effect of additionally added 1 µM levosimendan (Fig. 5A). This is in line with findings by others in human ventricular strips (Orstavik et al. 2014). Levosimendan increased also the positive (Fig. 5D) and negative (Fig. 5D) first derivatives of force of contraction versus time (dF/dt min and max). Levosimendan alone shortened concentration-dependently time to peak tension and time of relaxation (Fig. 5E). The positive inotropic effects of levosimendan (1 µM) were abrogated by 10 µM propranolol in human atrial preparations consistent with the action of levosimendan as a PDE III inhibitor in human atrial tissue (Fig. 5A). Interestingly and in contrast to mouse left atrial preparations, in human atrial preparations under the present experimental conditions, 10 µM rolipram failed to increase force of contraction (data not shown), consistent with the view that force of contraction in the human heart, at least in the atrium, is regulated not by PDE IV but mainly by PDE III. After maximal stimulation by 1 µM levosimendan, force of contraction was augmented by additionally applied EMD 57,033 (data not shown), a calcium sensitizer, suggesting that in human atrium, EMD 57,033 and levosimendan have different mechanisms of action (Fig. 1). A positive inotropic effect of EMD 57,033 in human atrial preparations has been reported before (Uhlmann et al. 1995) and EMD 57,033 was less effective than noradrenaline (Nankervis et al. 1994). Similarly, we had shown that the positive inotropic effect of levosimendan was lower than that of CGP 48,506 in muscle strips from failing human ventricles (Zimmermann et al. 1998). These data combined argue against a calcium-sensitizing effect of levosimendan, at least under our experimental conditions in the human atrium. Moreover, the maximum inotropic effect to levosimendan was lower than that of 10 µM isoprenaline (Fig. 5A, C), in line with reports in human ventricular preparations (Orstavik et al. 2014).

#### Phosphorylation in human atrial preparations

**Fig. 6:**
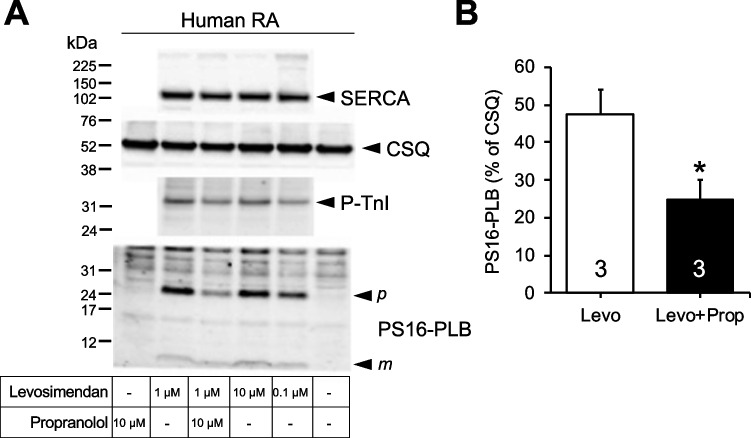
Levosimendan alone increases phosphorylation of phospholamban in the human heart. (**A**) Western blot of phosphorylated phospholamban at serine 16 (PS16-PLB) and the phosphorylated inhibitory subunit of troponin (P-TnI) in contracting human atrium. Horizontal arrows indicate the apparent molecular weight of phospholamban phosphorylated at serine 16 or phosphorylated inhibitory subunit of troponin (P-TnI), calsequestrin (CSQ), and SERCA were used as a loading control. Levosimendan 0.1 µM, 1 µM, or 10 µM or additional 10 µM propranolol was added to contracting human right atrial muscle strips, as indicated and freeze-clamped at the plateau of the inotropic effect. Controls for PLB phosphorylation were omission of drugs or application of propranolol alone. Apparent molecular weight standards are depicted in the left-hand first lane. Samples were not boiled (compare Fig. 4). Therefore, always a high (p) and low (m) molecular weight form of phospholamban is detectable. (**B**) Quantification of Western blots for PS16-PLB normalized to the CSQ signal as loading control. *p < 0.05 versus levosimendan (*t*-test, *n* = 3); Levo, levosimendan; Prop, propranolol

0.1 µM levosimendan, 1 µM levosimendan, and 10 µM levosimendan alone (that is, non cumulatively applied) increased the phosphorylation state of phospholamban at serine 16 and the phosphorylation state of the inhibitory subunit of troponin (TnI) in contracting human atrial strips that were freezed-clamped at the maximum of the positive inotropic effect (Fig. 6). The positive inotropic effects of levosimendan in human atrial strips were completely reversed by 10 µM propranolol (Fig. 6) as reported before in ventricular human muscle strips (Orstavik et al. 2014). In the same atrial preparations where force was recorded and that were freeze-clamped, 10 µM propranolol reduced the increase in the phosphorylation state of phospholamban and the inhibitory subunit of troponin induced by 1 µM levosimendan (Fig. 6), arguing against an action of levosimendan as a pure calcium sensitizer.

## Discussion

### Contractile data in mouse left atrium

Even if it was the primary aim to study the effects of levosimendan in human atrial preparations, we first studied mouse atrial preparations to understand this tissue in finer detail also in comparison to published animal models. The present data on the mouse left atrium are consistent with those in rat ventricular preparations by others (Orstavik et al. 2014). These authors like us noted that levosimendan when given alone was ineffective to raise force of contraction in rat ventricular muscle strips (Orstavik et al. 2014). Moreover, like in rat ventricle (Orstavik et al. 2014), cilostamide alone did not stimulate force of contraction in the mouse left atrial preparations. This lack of inotropic effect of cilostamide could be explained by the lower activity of phosphodiesterase III in the mouse heart compared to the human heart (for synopsis, see, for instance, table 2 in Neumann et al. 2019). In agreement with findings in rat papillary muscle (Orstavik et al. 2014), cilostamide repealed a positive inotropic effect of levosimendan in the mouse atrium. This observation is concordant with the interpretation that levosimendan heightens force of contraction in the mouse heart through repression of phosphodiesterase III. Another new finding, for the mouse heart, but in agreement with our findings and those of others in guinea pig cardiac preparations (Bokník et al. 1997; Edes et al. 1995) resides in the fact that levosimendan exalted the phosphorylation state of phospholamban. This resulted probably from a diminished activity of phosphodiesterases. Subsequently, levosimendan probably stepped up the levels of cAMP. This activated the cAMP-dependent protein kinase (Fig. 1). In this way, levosimendan probably boosted the phosphorylation state of phospholamban on serine 16.

In the rat, phosphodiesterase IV inhibition (by rolipram) or phosphodiesterase III inhibition (by milrinone or cilostamide) alone does not raise force of contraction or current through L-type currents in cardiomyocytes. Combining drugs that inhibit phosphodiesterase IV and phosphodiesterase III, however, augments force and L-type Ca^2+^ currents in rat cardiac preparations (Shahid and Nicholson 1990; Verde et al. 1999). The sum of phosphodiesterase III and phosphodiesterase IV activities might be so high in the rat ventricle that only if both phosphodiesterase III and phosphodiesterase IV are inhibited a hike in cAMP levels could take place. Thereby, force could be heightened (Shahid and Nicholson 1990). A similar reasoning can be made for the mouse heart.

The presence and extent of positive inotropic effects due to rolipram can be explained by species-dependent absolute or relative activities of phosphodiesterases: while rolipram hardly lowers the activity of phosphodiesterases I, II, and III (IC_50_ values = 100 µM to 1 mM), rolipram reduces phosphodiesterase IV potently (IC_50_ value = 1.1 µM) in guinea pig cardiac homogenates. However, rolipram up to 1 mM failed to increase force of contraction in guinea pig papillary muscles or the spontaneous beating rate in isolated guinea pig right atrial preparations (Bethke et al. 1993; Brunkhorst et al. 1989). Likewise, rolipram potently inhibited the activity of phosphodiesterase IV from the rat heart (with IC_50_ values around 1 µM). Nevertheless, rolipram alone (up to 100 µM) failed to increase force of contraction in rat isolated right ventricular papillary muscles (Shahid and Nicholson 1990). Hence, one explanation lies in the fact that the total activity of phosphodiesterase IV in rat cardiac samples is so small (Richter et al. 2011) that its inhibition remains inconsequential. Moreover, PDE isoforms are differentially expressed in the rat atria and ventricle that can explain different experimental results depending on the used tissue (Derici et al. 2019).

### Chronotropic effects of levosimendan in mouse right atrium

The present work clearly shows that levosimendan increased the beating rate in the mouse right atrium. This is in agreement with and extends our previous findings in the isolated guinea pig right atrium where we also noted an elevation of the beating rate by levosimendan. But that increase occurred in the absence of rolipram (Bokník et al. 1997). These positive chronotropic effects in mouse and guinea pig strongly argue against an action of levosimendan as a calcium sensitizer. At least in the guinea pig right atrium, a pure calcium sensitizer called CGP 48,506 did not alter the beating rate (Neumann et al. 1996). Moreover, the effect in the sinus node also seemed to result from amplification of cAMP levels. Endogenous noradrenaline may activate the β-adrenoceptor in the mouse sinus node. These findings are consistent with the view that levosimendan acts via elevation of cAMP also in sinus node cells. This observation concurs with the hypothesis that also in the mouse sinus node, levosimendan operates as a phosphodiesterase III inhibitor: fittingly, pre-incubation with cilostamide nullified the positive chronotropic effect of levosimendan.

### Effects of levosimendan in human atrium

In contrast to mouse atrial preparations, in isolated electrically stimulated human atrial preparations, levosimendan alone escalated force of contraction and shortened relaxation time and time to peak tension. We assume this is probably due to a surge in the phosphorylation state of phospholamban and of the phosphorylation state of the inhibitory subunit of troponin that we measured here. Cardiac relaxation is renowned to be enhanced by phosphorylation of phospholamban (Hamstra et al. 2020) and phosphorylation of the inhibitory subunit of troponin (Vetter et al. 2018). Positive inotropic effects of levosimendan in human atrial preparations have been delineated before by others (Sahin et al. 2007; Usta et al. 2004). However, they did not specify other contractile parameters like relaxation time (Sahin et al. 2007; Usta et al. 2004). On the other hand, our data are fostered by the fact that levosimendan shortened time of relaxation in human ventricular muscle strips (Orstavik et al. 2014).

In the human atrial preparations, no addition of rolipram was required to detect a positive inotropic effect of levosimendan. This resembles our findings in the guinea pig heart (Bokník et al. 1997) and those of the findings in human ventricular preparations (Orstavik et al. 2014). We assume from these facts that phosphodiesterase IV is not critical for force generation in the human heart, at least in our experimental setup. These data are not surprising as expression and/or function of PDE isoforms is different not only between left and right cavities of the heart or between atrium and ventricle but also between different species (an overview can be found in Calamera et al. (2022)). Therefore, data generated in, for example, rodents should be interpreted carefully because they cannot always be transferred directly to humans. Electrophysiological findings support this view: in isolated human atrial cardiomyocytes, levosimendan alone concentration-dependently (10 nM to 10 µM) elevated the L-type calcium current with an EC_50_ value of 54 nM, close to IC_50_ values referred for levosimendan in phosphodiesterases from the guinea pig heart, and the authors concluded “levosimendan may be the most potent phosphodiesterase inhibitor known” (Ajiro et al. 2002). Rolipram hardly impeded phosphodiesterases I, II, and III in human ventricular tissue but potently blocked phosphodiesterase IV (IC_50_ values: 3 µM (Reeves et al. 1987)). Our observation that rolipram alone did not exalt force of contraction in human atrial preparations is backed by findings in other laboratories (human atrium: 10 µM rolipram (Berk et al. 2016; Molina et al. 2012) and can be explained by the low relative activity of phosphodiesterase IV in the human heart (Table 2 in Neumann et al. 2019). Likewise, our observation that in the presence of cilostamide, rolipram was able to booster force of contraction, matched previous work of others in human atrial and ventricular preparations (Berk et al. 2016; Molenaar et al. 2013). Finally, our observation that propranolol countermands the positive inotropic effect of levosimendan in human atrial preparations also contradicts the prevailing view that levosimendan is a calcium sensitizer in the human heart.

A limitation of the study is that we were not able to measure cAMP or PDE activities in human cardiomyocytes. On the other hand, these measurements already have been down by others in rat and human cardiac samples (Orstavik et al. 2014; Ørstavik et al. 2015). It was clearly stated that levosimendan as well as its active metabolite inhibited PDE3 (Orstavik et al. 2014; Ørstavik et al. 2015). Therefore, we focused on the phosphorylation of phospholamban and troponin inhibitor as common pathway of cAMP increasing drugs. Besides, it would be very surprising to see a pure calcium sensitizer increasing phospholamban phosphorylation. A further limitation is that we would have liked to perform the experiments in human ventricular tissue, but on our institution, we could only get atrial human tissue. From a clinical point of view, force is mainly generated in the left ventricle, not in the right atrium. Therefore, our study has this limitation of which we are fully aware, but have to accept it.

## Conclusion

The present data strongly suggest that the positive inotropic and relaxant effects of levosimendan in the isolated human right atrium result from lowered activity of phosphodiesterase III. The contractile effects of levosimendan in the human atrium are caused, at least in part, by a booster of the phosphorylation state of phospholamban and the inhibitory subunit of troponin.

## Supplementary Information

Below is the link to the electronic supplementary material.

Supplementary file1 (PDF 623 KB)

## Data Availability

The data of this study are available from the corresponding author upon reasonable request.

## References

[CR1] Abi-Gerges A, Richter W, Lefebvre F, Mateo P, Varin A, Heymes C, Samuel J-L, Lugnier C, Conti M, Fischmeister R, Vandecasteele G (2009). Decreased expression and activity of cAMP phosphodiesterases in cardiac hypertrophy and its impact on β-adrenergic cAMP signals. Circ Res.

[CR2] Ajiro Y, Hagiwara N, Katsube Y, Sperelakis N, Kasanuki H (2002). Levosimendan increases L-type Ca2+ current via phosphodiesterase-3 inhibition in human cardiac myocytes. Eur J Pharmacol.

[CR3] Berk E, Christ T, Schwarz S, Ravens U, Knaut M, Kaumann AJ (2016). In permanent atrial fibrillation, PDE3 reduces force responses to 5-HT, but PDE3 and PDE4 do not cause the blunting of atrial arrhythmias. Br J Pharmacol.

[CR4] Bethke T, Meyer W, Schmitz W, Scholz H, Wenzlaff H, Armah BI, Brückner R, Raap A (1993). High selectivity for inhibition of phosphodiesterase III and positive inotropic effects of MCI-154 in guinea pig myocardium. J Cardiovasc Pharmacol.

[CR5] Bokník P, Neumann J, Kaspareit G, Schmitz W, Scholz H, Vahlensieck U, Zimmermann N (1997). Mechanisms of the contractile effects of levosimendan in the mammalian heart. J Pharmacol Exp Ther.

[CR6] Boknik P, Drzewiecki K, Eskandar J, Gergs U, Grote-Wessels S, Fabritz L, Kirchhof P, Müller FU, Stümpel F, Schmitz W, Zimmermann N, Kirchhefer U, Neumann J (2018). Phenotyping of mice with heart specific overexpression of A2A-adenosine receptors: evidence for cardioprotective effects of A2A-adenosine receptors. Front Pharmacol.

[CR7] Brunkhorst D, der Leyen H, Meyer W, Nigbur R, Schmidt-Schumacher C, Scholz H (1989). Relation of positive inotropic and chronotropic effects of pimobendan, UD-CG 212 Cl, milrinone and other phosphodiesterase inhibitors to phosphodiesterase III inhibition in guinea-pig heart. Naunyn Schmiedebergs Arch Pharmacol.

[CR8] Calamera G, Moltzau LR, Levy FO, Andressen KW (2022). Phosphodiesterases and compartmentation of cAMP and cGMP signaling in regulation of cardiac contractility in normal and failing hearts. Int J Mol Sci.

[CR9] Danielsen W, der Leyen H, Meyer W, Neumann J, Schmitz W, Scholz H, Starbatty J, Stein B, Döring V, Kalmár P (1989). Basal and isoprenaline-stimulated cAMP content in failing versus nonfailing human cardiac preparations. J Cardiovasc Pharmacol.

[CR10] Derici MK, Sadi G, Cenik B, Güray T, Demirel-Yilmaz E (2019). Differential expressions and functions of phosphodiesterase enzymes in different regions of the rat heart. Eur J Pharmacol.

[CR11] Edes I, Kiss E, Kitada Y, Powers FM, Papp JG, Kranias EG, Solaro RJ (1995). Effects of Levosimendan, a cardiotonic agent targeted to troponin C, on cardiac function and on phosphorylation and Ca2+ sensitivity of cardiac myofibrils and sarcoplasmic reticulum in guinea pig heart. Circ Res.

[CR12] Endoh M (2015). Does levosimendan act as a Ca2+ sensitizer or PDE3 inhibitor?: Commentary on Orstavik et al., Br J Pharmacol 171: 5169–5181. Br J Pharmacol.

[CR13] Feldman MD, Copelas L, Gwathmey JK, Phillips P, Warren SE, Schoen FJ, Grossman W, Morgan JP (1987). Deficient production of cyclic AMP: pharmacologic evidence of an important cause of contractile dysfunction in patients with end-stage heart failure. Circulation.

[CR14] Galindo-Tovar A, Kaumann AJ (2008). Phosphodiesterase-4 blunts inotropism and arrhythmias but not sinoatrial tachycardia of (-)-adrenaline mediated through mouse cardiac beta(1)-adrenoceptors. Br J Pharmacol.

[CR15] Gergs U, Boknik P, Schmitz W, Simm A, Silber R-E, Neumann J (2009). A positive inotropic effect of adenosine in cardiac preparations of right atria from diseased human hearts. Naunyn Schmiedebergs Arch Pharmacol.

[CR16] Gergs U, Böckler A, Ebelt H, Hauptmann S, Keller N, Otto V, Pönicke K, Schmitz W, Neumann J (2013). Human 5-HT_4_ receptor stimulation in atria of transgenic mice. Naunyn Schmiedebergs Arch Pharmacol.

[CR17] Gergs U, Fahrion CM, Bock P, Fischer M, Wache H, Hauptmann S, Schmitz W, Neumann J (2017). Evidence for a functional role of calsequestrin 2 in mouse atrium. Acta Physiol (oxf).

[CR18] Gergs U, Trapp T, Bushnaq H, Simm A, Silber R-E, Neumann J (2019). Age-dependent protein expression of serine/threonine phosphatases and their inhibitors in the human cardiac atrium. Adv Med.

[CR19] Gergs U, Bernhardt G, Buchwalow IB, Edler H, Fröba J, Keller M, Kirchhefer U, Köhler F, Mißlinger N, Wache H, Neumann J (2019). Initial characterization of transgenic mice overexpressing human histamine H2 receptors. J Pharmacol Exp Ther.

[CR20] Haikala H (1995). Cardiac troponin C as a target protein for a novel calcium sensitizing drug, levosimendan. J Mol Cell Cardiol.

[CR21] Haikala H (1997). The role of cAMP- and cGMP-dependent protein kinases in the cardiac actions of the new calcium sensitizer, levosimendan. Cardiovasc Res.

[CR22] Hamstra SI, Whitley KC, Baranowski RW, Kurgan N, Braun JL, Messner HN, Fajardo VA (2020). The role of phospholamban and GSK3 in regulating rodent cardiac SERCA function. Am J Physiol Cell Physiol.

[CR23] Hasenfuss G, Pieske B, Castell M, Kretschmann B, Maier LS, Just H (1998). Influence of the novel inotropic agent levosimendan on isometric tension and calcium cycling in failing human myocardium. Circulation.

[CR24] Kaheinen P, Pollesello P, Hertelendi Z, Borbély A, Szilágyi S, Nissinen E, Haikala H, Papp Z (2006). Positive inotropic effect of levosimendan is correlated to its stereoselective Ca2+-sensitizing effect but not to stereoselective phosphodiesterase inhibition. Basic Clin Pharmacol Toxicol.

[CR25] Maack C, Eschenhagen T, Hamdani N, Heinzel FR, Lyon AR, Manstein DJ, Metzger J, Papp Z, Tocchetti CG, Yilmaz MB, Anker SD, Balligand J-L, Bauersachs J, Brutsaert D, Carrier L, Chlopicki S, Cleland JG, de Boer RA, Dietl A, Fischmeister R, Harjola V-P, Heymans S, Hilfiker-Kleiner D, Holzmeister J, de Keulenaer G, Limongelli G, Linke WA, Lund LH, Masip J, Metra M, Mueller C, Pieske B, Ponikowski P, Ristić A, Ruschitzka F, Seferović PM, Skouri H, Zimmermann WH, Mebazaa A (2019). Treatments targeting inotropy. Eur Heart J.

[CR26] Molenaar P, Christ T, Hussain RI, Engel A, Berk E, Gillette KT, Chen L, Galindo-Tovar A, Krobert KA, Ravens U, Levy FO, Kaumann AJ (2013). PDE3, but not PDE4, reduces β_1_ - and β_2_-adrenoceptor-mediated inotropic and lusitropic effects in failing ventricle from metoprolol-treated patients. Br J Pharmacol.

[CR27] Molina CE, Leroy J, Richter W, Xie M, Scheitrum C, Lee I-O, Maack C, Rucker-Martin C, Donzeau-Gouge P, Verde I, Llach A, Hove-Madsen L, Conti M, Vandecasteele G, Fischmeister R (2012). Cyclic adenosine monophosphate phosphodiesterase type 4 protects against atrial arrhythmias. J Am Coll Cardiol.

[CR28] Nankervis R, Lues I, Brown L (1994). Calcium sensitization as a positive inotropic mechanism in diseased rat and human heart. J Cardiovasc Pharmacol.

[CR29] Neumann J, Scholz H, Döring V, Schmitz W, Meyerinck L, Kalmár P (1988). Increase in myocardial Gi-proteins in heart failure. The Lancet.

[CR30] Neumann J, Bokník P, Schmitz W, Scholz H, Zimmermann N (1995). Comparison of the stereoselective effects of a thiadiazinone derivative on contractile parameters and protein phosphorylation in the mammalian ventricle. J Cardiovasc Pharmacol.

[CR31] Neumann J, Eschenhagen T, Grupp IL, Haverich A, Herzig JW, Hirt S, Kalmár P, Schmitz W, Scholz H, Stein B, Wenzlaff H, Zimmermann N (1996). Positive inotropic effects of the calcium sensitizer CGP 48506 in failing human myocardium. J Pharmacol Exp Ther.

[CR32] Neumann J, Boknik P, Matherne GP, Lankford A, Schmitz W (2003). Pertussis toxin sensitive and insensitive effects of adenosine and carbachol in murine atria overexpressing A(1)-adenosine receptors. Br J Pharmacol.

[CR33] Neumann J, Käufler B, Gergs U (2019). Which phosphodiesterase can decrease cardiac effects of 5-HT4 receptor activation in transgenic mice?. Naunyn Schmiedebergs Arch Pharmacol.

[CR34] Neumann J, Seidler T, Fehse C, Marušáková M, Hofmann B, Gergs U (2021). Cardiovascular effects of metoclopramide and domperidone on human 5-HT4-serotonin-receptors in transgenic mice and in human atrial preparations. Eur J Pharmacol.

[CR35] Neumann J, Voss R, Laufs U, Werner C, Gergs U (2021). Phosphodiesterases 2, 3 and 4 can decrease cardiac effects of H2-histamine-receptor activation in isolated atria of transgenic mice. Naunyn Schmiedebergs Arch Pharmacol.

[CR36] Orstavik O, Ata SH, Riise J, Dahl CP, Andersen GØ, Levy FO, Skomedal T, Osnes J-B, Qvigstad E (2014). Inhibition of phosphodiesterase-3 by levosimendan is sufficient to account for its inotropic effect in failing human heart. Br J Pharmacol.

[CR37] Ørstavik Ø, Manfra O, Andressen KW, Andersen GØ, Skomedal T, Osnes J-B, Levy FO, Krobert KA (2015). The inotropic effect of the active metabolite of levosimendan, OR-1896, is mediated through inhibition of PDE3 in rat ventricular myocardium. PLoS ONE.

[CR38] Packer M, Carver JR, Rodeheffer RJ, Ivanhoe RJ, DiBianco R, Zeldis SM, Hendrix GH, Bommer WJ, Elkayam U, Kukin ML (1991). Effect of oral milrinone on mortality in severe chronic heart failure. The PROMISE Study Research Group. N Engl J Med.

[CR39] Papp Z, Agostoni P, Alvarez J, Bettex D, Bouchez S, Brito D, Černý V, Comin-Colet J, Crespo-Leiro MG, Delgado JF, Édes I, Eremenko AA, Farmakis D, Fedele F, Fonseca C, Fruhwald S, Girardis M, Guarracino F, Harjola V-P, Heringlake M, Herpain A, Heunks LM, Husebye T, Ivancan V, Karason K, Kaul S, Kivikko M, Kubica J, Masip J, Matskeplishvili S, Mebazaa A, Nieminen MS, Oliva F, Papp J-G, Parissis J, Parkhomenko A, Põder P, Pölzl G, Reinecke A, Ricksten S-E, Riha H, Rudiger A, Sarapohja T, Schwinger RH, Toller W, Tritapepe L, Tschöpe C, Wikström G, von Lewinski D, Vrtovec B, Pollesello P (2020). Levosimendan Efficacy and Safety: 20 years of SIMDAX in Clinical Use. Card Fail Rev.

[CR40] Reeves ML, Leigh BK, England PJ (1987). The identification of a new cyclic nucleotide phosphodiesterase activity in human and guinea-pig cardiac ventricle. Implications for the mechanism of action of selective phosphodiesterase inhibitors. Biochem J.

[CR41] Richter W, Xie M, Scheitrum C, Krall J, Movsesian MA, Conti M (2011). Conserved expression and functions of PDE4 in rodent and human heart. Basic Res Cardiol.

[CR42] Rüegg JC, Pfitzer G, Eubler D, Zeugner C (1984). Effect on contractility of skinned fibres from mammalian heart and smooth muscle by a new benzimidazole derivative, 4,5-dihydro-6-2-(4-methoxyphenyl)-1H-benzimidazol-5-yl-5-methy l-3(2H )- pyridazinone. Arzneimittelforschung.

[CR43] Sahin AS, Görmüş N, Duman A (2007). Preconditioning with levosimendan prevents contractile dysfunction due to H2O2-induced oxidative stress in human myocardium. J Cardiovasc Pharmacol.

[CR44] Schmitz W, von der Leyen H, Meyer W, Neumann J, Scholz H (1989). Phosphodiesterase inhibition and positive inotropic effects. J Cardiovasc Pharmacol.

[CR45] Schmitz W, Eschenhagen T, Mende U, Müller FU, Neumann J, Scholz H (1992). Phosphodiesterase inhibition and positive inotropy in failing human myocardium. Basic Res Cardiol.

[CR46] Scholz H, Meyer W (1986). Phosphodiesterase-inhibiting properties of newer inotropic agents. Circulation.

[CR47] Shahid M, Nicholson CD (1990). Comparison of cyclic nucleotide phosphodiesterase isoenzymes in rat and rabbit ventricular myocardium: positive inotropic and phosphodiesterase inhibitory effects of Org 30029, milrinone and rolipram. Naunyn Schmiedebergs Arch Pharmacol.

[CR48] Sucharov CC, Nakano SJ, Slavov D, Schwisow JA, Rodriguez E, Nunley K, Medway A, Stafford N, Nelson P, McKinsey TA, Movsesian M, Minobe W, Carroll IA, Taylor MRG, Bristow MR (2019). A PDE3A promoter polymorphism regulates cAMP-induced transcriptional activity in failing human myocardium. J Am Coll Cardiol.

[CR49] Uhlmann R, Schwinger RH, Lues I, Erdmann E (1995). EMD 53998 acts as Ca(2+)-sensitizer and phosphodiesterase III-inhibitor in human myocardium. Basic Res Cardiol.

[CR50] Usta C, Puddu PE, Papalia U, de Santis V, Vitale D, Tritapepe L, Mazzesi G, Miraldi F, Ozdem SS (2004). Comparision of the inotropic effects of levosimendan, rolipram, and dobutamine on human atrial trabeculae. J Cardiovasc Pharmacol.

[CR51] Ventura C, Miller R, Wolf HP, Beier N, Jonas R, Klockow M, Lues I, Hano O, Spurgeon HA, Lakatta EG (1992). Novel diazinone derivatives separate myofilament Ca2+ sensitization and phosphodiesterase III inhibitory effects in guinea pig myocardium. Circ Res.

[CR52] Verde I, Vandecasteele G, Lezoualc'h F, Fischmeister R (1999). Characterization of the cyclic nucleotide phosphodiesterase subtypes involved in the regulation of the L-type Ca2+ current in rat ventricular myocytes. Br J Pharmacol.

[CR53] Vetter AD, Houang EM, Sell JJ, Thompson BR, Sham YY, Metzger JM (2018). TnI structural interface with the N-terminal lobe of TnC as a determinant of cardiac contractility. Biophys J.

[CR54] Virág L, Hála O, Marton A, Varró A, Papp JG (1996). Cardiac electrophysiological effects of levosimendan, a new calcium sensitizer. Gen Pharmacol Vasc Syst.

[CR55] von der Leyen H, Mende U, Meyer W, Neumann J, Nose M, Schmitz W, Scholz H, Starbatty J, Stein B, Wenzlaff H (1991). Mechanism underlying the reduced positive inotropic effects of the phosphodiesterase III inhibitors pimobendan, adibendan and saterinone in failing as compared to nonfailing human cardiac muscle preparations. Naunyn Schmiedebergs Arch Pharmacol.

[CR56] Zimmermann N, Bokník P, Gams E, Herzig JW, Neumann J, Schmitz W, Scholz H, Wenzlaff H (1996). Positive inotropic effects of the calcium sensitizer CGP 48506 in guinea pig myocardium. J Pharmacol Exp Ther.

[CR57] Zimmermann N, Boknik P, Gams E, Herzig JW, Neumann J, Scholz H (1998). Calcium sensitization as new principle of inotropic therapy in end-stage heart failure?1. Eur J Cardiothorac Surg.

