# Supplementary material for: Levosimendan increases the phosphorylation state of phospholamban in the isolated human atrium
Source: Naunyn Schmiedebergs Arch Pharmacol. 2022 Nov 29;396(4):669–82. doi: 10.1007/s00210-022-02348-7 (PMC10042762; doi:10.1007/s00210-022-02348-7)
Supplement: Supplementary file 1 — Supplementary file1 (PDF 623 KB) [file 210_2022_2348_MOESM1_ESM.pdf]

Original Western blots

1. Ponceau red staining

Mouse samples Human samples

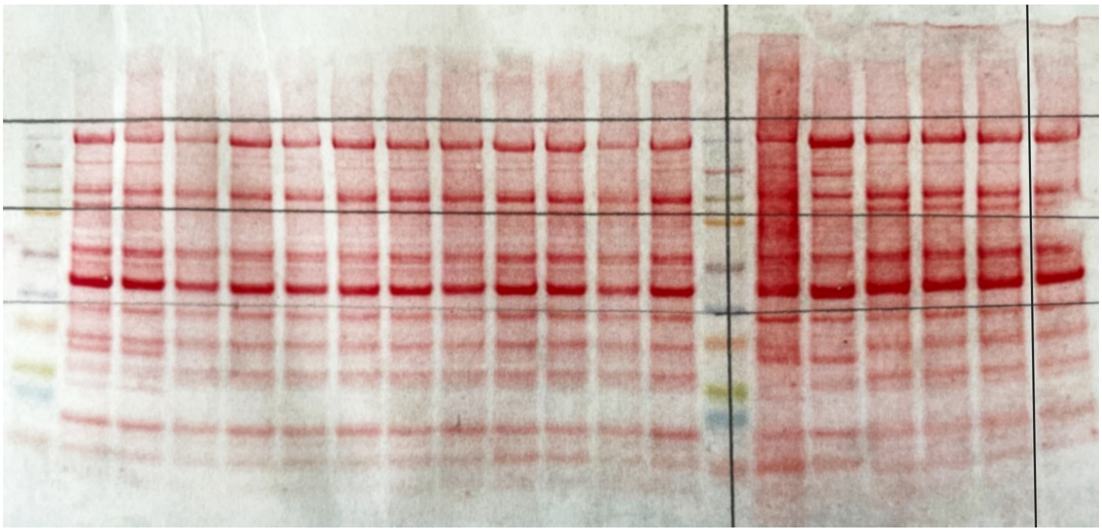

PS16-PLB

SERCA

CSQ

P-TnI

The lines on the ponceau stained blot show where the membrane was cutted before antibody incubation.

2. Immunoblotting (in order to safe money, only the regions of interest were incubated with antibodies)

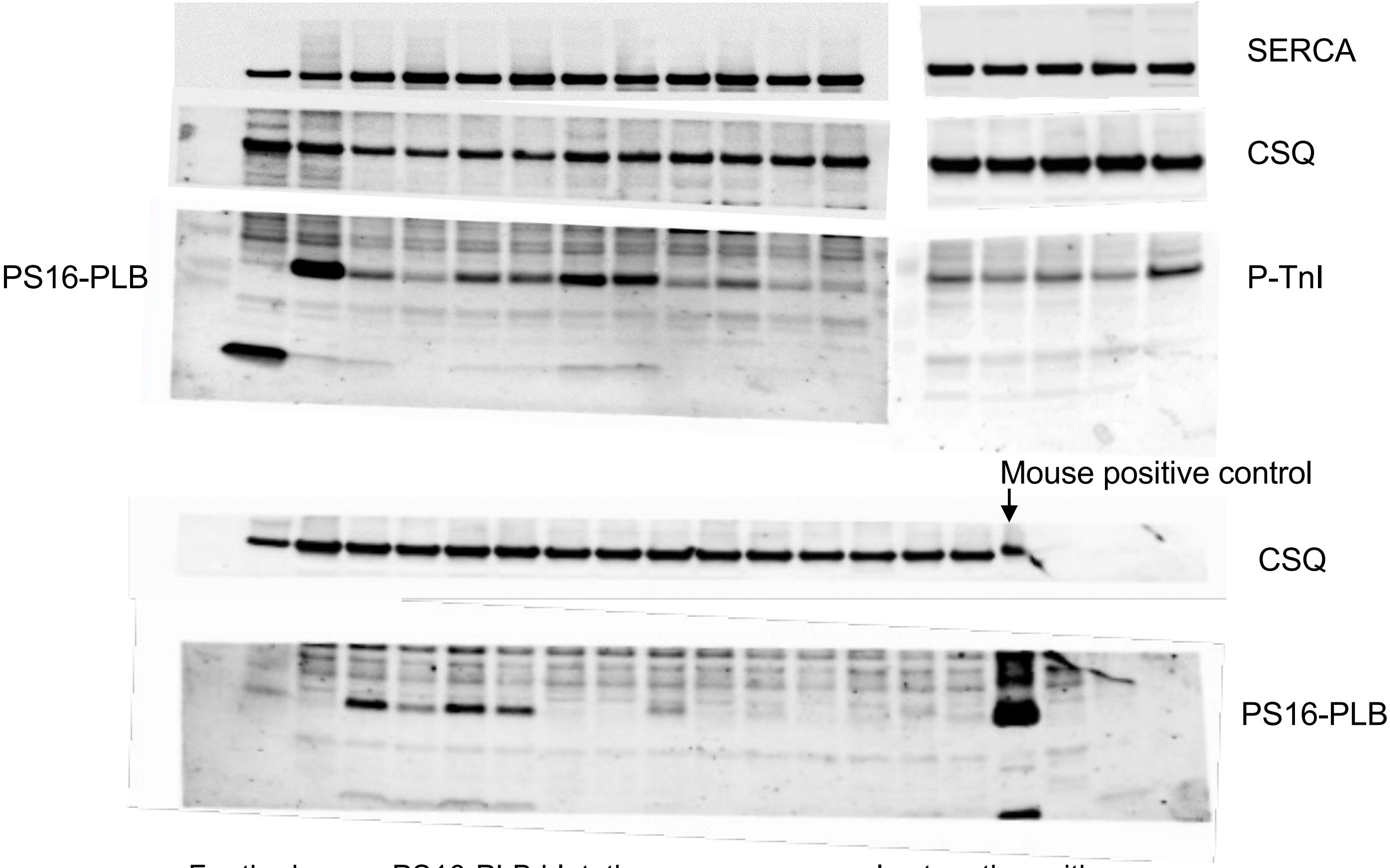

PS16-PLB

SERCA

CSQ

P-TnI

CSQ

PS16-PLB

For the human PS16-PLB blot, the very same samples together with new samples and controls were run on a different gel.
